# Paradoxical Improvement in Malignant Pleural Mesothelioma Outcomes Following Delayed Treatment Initiation

**DOI:** 10.3390/cancers16223755

**Published:** 2024-11-07

**Authors:** Ashwin Kulshrestha, Emanuela Taioli, Andrea Wolf, Raja Flores, Stephanie Tuminello

**Affiliations:** 1 Icahn School of Medicine at Mount Sinai, New York, NY 10029, USA; ashwin.kulshrestha@icahn.mssm.edu; 2 Institute for Translational Epidemiology, Icahn School of Medicine at Mount Sinai, New York, NY 10029, USA; 3 Department of Thoracic Surgery, Icahn School of Medicine at Mount Sinai, New York, NY 10029, USA; 4 Tisch Cancer Institute, Icahn School of Medicine at Mount Sinai, New York, NY 10029, USA

**Keywords:** malignant pleural mesothelioma (MPM), time to treatment initiation (TTI), thoracic surgery, disparities, survival

## Abstract

Background/Objectives: Time to treatment initiation (TTI) has been identified as a predictor of survival in many cancers, but its impact on malignant pleural mesothelioma (MPM) is unknown. This study investigates factors influencing TTI in MPM and its association with overall survival. Methods: The Surveillance, Epidemiology, and End Results (SEER) database was used to obtain data for MPM patients in the United States. TTI was defined as the number of days from diagnosis to initiation of first treatment, and delayed TTI was defined as exceeding the median TTI. Χ^2^ tests and *t*-tests compared sociodemographic and clinical differences between early and delayed TTI groups, while Kaplan–Meier and Cox proportional hazards models evaluated relationships between prognostic factors, TTI, and survival. Results: Among 4879 MPM patients, the median TTI was 39 days. Median survival was 10 months among early TTI patients and 13 months among delayed TTI patients. Patients with epithelioid histology were more likely to have delayed TTI, as were patients who received combination therapy or were diagnosed more recently (*p* < 0.0001). Adjusting for covariates, delayed TTI status remained associated with better survival (HR 0.79, 95% CI: 0.74–0.84). Conclusions: This study presents an important insight into the management of MPM, demonstrating that delayed time to treatment initiation is positively associated with improved overall survival, contrary to findings in most cancers. This finding underscores the importance of comprehensive, multidisciplinary care, as delays due to robust staging evaluations and patient travel to high-volume centers of excellence likely contribute to delays in treatment. Taken together, these results suggest that clinicians should prioritize personalized treatment planning and collaborative care over a push to rapidly initiate treatment to optimize patient outcomes in MPM.

## Simple Summary

We analyzed records from nearly 5000 patients diagnosed with malignant pleural mesothelioma (MPM) to understand how time from diagnosis to initiation of treatment (called Time to Treatment Initiation, or TTI) affects survival. Surprisingly, we found that patients who began treatment later (after a median of 39 days) had better overall survival compared to those who started treatment earlier. Specifically, patients with delayed treatment lived a median of 13 months, while those with earlier treatment lived 10 months. This result is surprising and contradicts what is known in most cancers, which is that quicker treatment typically leads to better outcomes. This may be due to the need for specialized care in MPM, including thorough evaluations for proper treatment decision-making and travel to expert medical centers. These findings suggest that for MPM, taking more time to carry out a carefully selected, multidisciplinary plan may be the optimal approach for patient survival.

## 1. Introduction

Malignant pleural mesothelioma (MPM) is a rare but aggressive cancer originating in the mesothelial cells of the pleura, with a median overall survival of approximately 10 months following diagnosis [1]. The onset of MPM is classically linked to asbestos exposure, and the incidence of MPM has been slightly declining in industrialized nations where the use of asbestos has been severely restricted or prohibited [2]. However, the average latency period between exposure to asbestos and the onset of disease is approximately 40 years, and often, the impact of asbestos regulation on incidence is not appreciable for several decades [2,3]. In developing nations with continued use of asbestos, the incidence of mesothelioma is expected to continue increasing, with recent data suggesting that the percentage of cases among women worldwide may be growing due to continued unknown environmental or occupational exposures to asbestos [3]. In short, the disease burden of MPM is heterogeneous across the globe but is largely expected to increase in the coming decades.

The optimal treatment approach for malignant pleural mesothelioma remains contentious in the absence of a unanimous standard of care, but major committee guidelines largely support the use of multimodal approaches when available and indicated [4,5,6]. Cancer-directed surgery has been shown to be associated with better survival, but data indicates that only 22% of patients undergo surgery for MPM [7]. The two surgical approaches—extended pleurectomy-decortication (P/D) and extrapleural pneumonectomy—are often supplemented with chemotherapy and/or radiation in suitable patients. Overall, applying a surgery-based multimodal approach has been associated with a 15% 5-year survival rate [7]. Recent advances in treatment options include a first-line approval of nivolumab plus ipilimumab, with ongoing trials evaluating potential immunotherapy combinations [5,8]. Moving forward, proper treatment allocation will be increasingly essential in facilitating multimodal care in mesothelioma. However, despite the growing availability of treatment options, the prognosis for patients with MPM remains poor.

Research into trends in diagnosis, treatment, and survival has revealed marked disparities across MPM patient groups. Of the three primary histological subtypes—epithelioid, fibrous, and biphasic—the percentage of epithelioid differentiation has been reported to independently be a predictor of better survival [9]. Female patients are more likely to present at a younger age and with epithelioid histology and have been shown to have a survival advantage over male patients [10]. Conversely, Black patients have been found to have significantly poorer survival compared to White patients [11]. Higher-income patients have also been shown to have significantly better overall survival compared to their counterparts [11]. However, the totality of factors driving disparate outcomes in patients with MPM remains unclear, and further study of contributing factors is necessary given the poor outcomes of the disease.

The time between diagnosis of cancer and treatment initiation, henceforth simply referred to as time to treatment initiation (TTI), has been identified as a significant mediator of overall survival in many cancer types. In a study of over 2 million patients with the four most common types of cancer in the United States—breast, prostate, non–small cell lung cancer (NSCLC), and colon—delayed TTI was shown to be associated with increased 5- and 10-year mortality rates [12]. Similar findings have been reported for head and neck cancers, bladder cancer, and glioma, among others [13,14,15]. In NSCLC specifically, stage 1 and 2 patients with a TTI over 45 days were found to have significant increases in mortality compared to patients with a TTI under 45 days. Given this profound impact, a TTI ≤ 45 days has been suggested as a clinically targetable timeframe for initiation of NSCLC treatment [16].

Notably, TTI has been reported to vary significantly by patient demographics, representing an additional contributing factor to ongoing health disparities in cancer treatment and survival. Women of racial and ethnic minorities receiving surgery at a comprehensive breast cancer center were found to have consistent delays in treatment, with Black women having the greatest delay—1.42 times longer than White patients—after adjusting for patient and disease characteristics [17]. In prostate cancer, uninsured patients or patients with Medicaid were found to have higher odds of delayed treatment initiation compared to patients with private insurance or Medicare [18]. Black patients diagnosed with anal squamous cell cancer were reported to have greater odds of treatment delay, which was subsequently found to be independently associated with poorer overall survival even after controlling for other prognostic variables [19].

Given the aggressive nature of MPM, there is a sensed urgency to initiate treatment as rapidly as possible. However, neither the factors impacting TTI in patients with MPM—nor the impact of delayed TTI on survival—is currently known. The present study sought to examine the clinical and sociodemographic factors that impact TTI in patients diagnosed with MPM. Furthermore, we aimed to determine whether increased TTI among MPM patients is associated with poorer overall survival. We hypothesized that TTI may be a yet-unreported driver of recognized heterogeneity in outcomes among patients diagnosed with MPM.

## 2. Materials and Methods

### 2.1. Study Population

**Figure 1:**
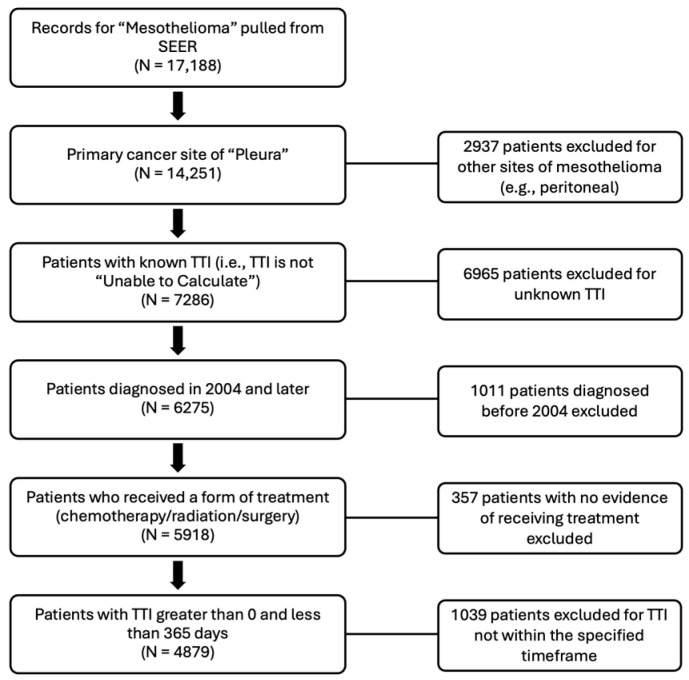
Patient selection flowchart (*n* = 4879).

The National Cancer Institute’s Surveillance, Epidemiology, and End Results (SEER) database is a publicly available, de-identified dataset containing detailed information on incident cancer cases throughout the United States, with registries covering approximately 37 percent of the population [20]. The SEER database was queried for all records from 2000 to 2021 containing an ICD-O-3 site recode of “Mesothelioma” (*n* = 17,188) [20]. Autopsy cases were excluded, and the sample was further restricted to patients with pathologically proven mesothelioma of the pleura (*n*_excluded_ = 2937), omitting less common sites of mesothelial malignancy (peritoneum, pericardium, mediastinum, etc.). Patients with an unknown TFTI were also excluded (*n*_excluded_ = 6965). Additionally, those who were diagnosed more than 20 years ago (i.e., prior to 2004) were excluded to ensure the sample was most representative of current trends in diagnosis and management while providing a large enough sample to power such an analysis (*n*_excluded_ = 1011). Patients who were not known to have received any treatment were also excluded from the analysis (*n***_excluded_** = 357). Finally, patients with a TTI of 0 days were excluded, as this likely reflects initiation on active surveillance, or greater than 365 days, as this likely reflects the outcome of non-clinical, demographic, or prognostic factors (*n*_excluded_ = 1039). Patients with missing or unknown data for all other sociodemographic or clinical variables (e.g., SEER stage, marital status, etc.) were not excluded from the sample (Figure 1).

### 2.2. Variables

Time to treatment initiation (TTI) data are provided directly by SEER and are calculated prior to deidentification as the time from diagnosis to the initiation of first treatment. Treatments recognized in this calculation include chemotherapy, radiation therapy, hormonal therapy, immunotherapy, surgery, and active surveillance [20]. For patients who received a combination therapy, their TTI data reflects the time to initiation of the first therapy in the sequence. Delayed TTI was defined as a duration exceeding the median TTI.

Individual treatment variables in SEER were used to create a composite variable describing the totality of treatment(s) a patient was known to receive. For example, if they had “Yes” for chemotherapy and radiation and “No/Unknown” for surgery, they would be listed as having received “Systemic Only”; if a patient had “Yes” for chemotherapy, in addition to a form of radiation therapy and surgery, they would be listed as having received “Combination Treatment”. In addition to type of treatment received, information on sociodemographic characteristics, such as race (Black, White, or Other/Unknown), age, sex (male, female), household income status (<$60,000, $60,000–$99,999, >$100,000), marital status (married, single, other/unknown), as well as disease characteristics, including stage (localized, regional, distant, unknown/unstaged), histology (mesothelioma, NOS; epithelioid; fibrous or sarcomatoid; biphasic or mixed), laterality (left, right, bilateral/unknown), plus practice setting (urban, suburban/rural) and year of diagnosis were extracted from the SEER dataset.

### 2.3. Statistical Analysis

We compared patients with or without delayed TTI (TTI above or below the median, respectively). Baseline demographic and clinical characteristics were compared using χ^2^ tests for categorical variables and *t*-tests for continuous variables, as these choices of statistical tests were appropriate given the data distributions. Overall survival was defined as the duration between diagnosis and either death or last follow-up. Further, Kaplan–Meier survival curves were created to compare probabilities of survival over time across strata. Univariate and multivariate Cox proportional hazards models were generated to determine the independent and adjusted contributions of predictors on TTI and overall survival. Assumptions of this model were met, and the proportionality of hazards was confirmed. Models were adjusted for age, sex, race, income, stage, histology, treatment, practice setting, and year of diagnosis. Additional sensitivity analyses examined the association between TTI and survival in three subsamples: patients who received surgery only, patients with epithelioid histology only, and patients with localized or regional staging only. χ^2^ tests, *t*-tests, and Cox proportional hazards models were applied for these sensitivity analyses as well. Analysis was performed in SAS version 9.4. This protocol was determined to be exempt from review by the Mount Sinai Institutional Review Board and Program for the Protection of Human Subjects (PRMC#: 24-630).

## 3. Results

The median time from diagnosis to treatment initiation among the 4879 patients included in the overall analysis was 39 days. Significant differences were observed among patients in the early and delayed TTI groups (*n* = 2428, 50% & *n* = 2451, 50%, respectively) (Table 1). Delayed TTI patients were more likely to have epithelioid histology (*p* < 0.0001). A significantly greater percentage of patients in the early TTI group received surgery as their only form of treatment, whereas a greater percentage of patients in the delayed TTI group received a combination therapy regimen (*p* < 0.0001). Variation in TTI was also observed over time (*p* < 0.0001). The majority of patients diagnosed prior to 2013 had an early TTI (1357 vs. 1225), whereas the majority of patients diagnosed since 2013 had a delayed TTI (1226 vs. 1071). The incidence data from 2019 to 2022 shows a reduced incidence of disease compared to prior years, even when accounting for the smaller range of years included. No significant differences in age, sex, race, practice setting, income, and marital status were observed between the two TTI classes.

**Figure 2:**
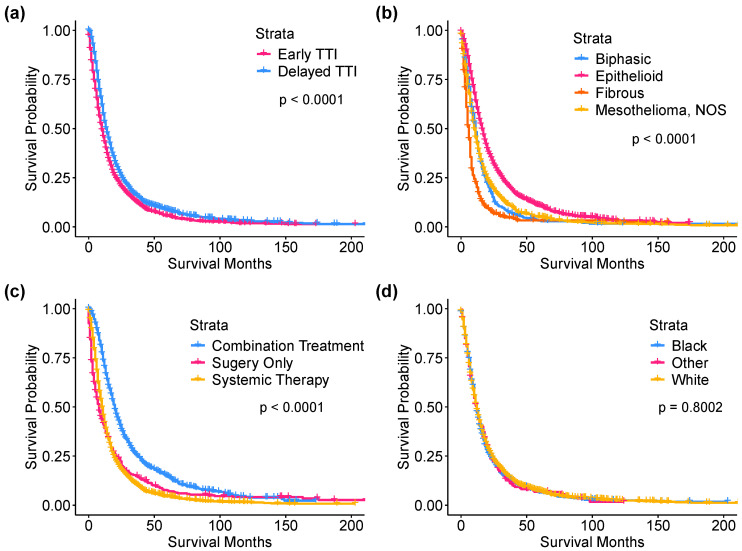
Survival probability over time by strata. Median survival: (**a**) Delayed TTI: 13 months, Early TTI: 10 months (*p* < 0.0001); (**b**) Combination therapy: 18.5 months, Systemic therapy only: 10 months, Surgery only: 7 months (*p* < 0.0001); (**c**) Biphasic histology: 11 months, Epithelioid histology: 15 months, Fibrous (sarcomatoid) histology: 6 months, Other/unspecified histology: 10 months (*p* < 0.0001); (**d**) Black: 11 months, Other: 11 months, White: 11 months (*p* = 0.8002).

The median OS for the entire study cohort was 11 months (Supplemental Table S1). Median survival among the early TTI group was 10 months, compared to 13 months in the delayed TTI group (*p* < 0.0001). This effect persisted after adjusting for covariates (HR_adj_: 0.79, 95% CI: 0.74–0.84) (Table 2). Several factors were statistically significantly associated with overall survival (Figure 2). Increased age was associated with a modest increase in the hazard of death (HR_adj_: 1.02, 95% CI: 1.01–1.02 per each year increase in age), whereas more recent year of diagnosis was associated with a modest decrease (HR_adj_: 0.98, 95% CI: 0.97–0.99). Male patients were found to have poorer survival (HR_adj_: 1.19, 95% CI: 1.10–1.28), as were Black patients when compared to White patients (HR_adj_: 1.12, 95% CI: 1.03–1.22). Epithelioid histology was associated with better survival (HR_adj_: 0.78, 95% CI: 0.73–0.84), whereas the association with fibrous and biphasic histologies was in the opposite direction (HR_adj_: 1.83, 95% CI: 1.65–2.04; HR_adj_: 1.26, 95% CI: 1.13–1.40, respectively). Compared to patients who were treated with surgery only, there was no significant difference in survival for patients who received only systemic treatment (HR_adj_: 0.97, 95% CI: 0.86–1.08). However, receipt of combination therapy was associated with significantly better overall survival compared to receipt of surgery alone (HR_adj_: 0.62, 95% CI: 0.54–0.70). Regarding SEER stage, both regional and distant staging were associated with poorer overall survival compared to localized staging (HR_adj_: 1.16, 95% CI: 1.03–1.32; HR_adj_: 1.39, 95% CI: 1.24–1.56, respectively).

### Sensitivity Analyses

The positive association between delayed TTI and overall survival persisted in three sensitivity analyses that were performed: first on patients treated with surgery alone (Supplemental Table S2), a second on patients with epithelioid histology only (Supplemental Table S3), and a third on patients with locoregional disease only (Supplemental Table S4).

For patients treated with surgery alone (*n* = 392), median overall survival in the early TTI group was 4 months, compared to 11 months in the delayed TTI group (*p* = 0.0003; median TTI of 34.5 days). The two groups varied significantly only in histology and practice settings, with delayed TTI patients being more likely to have epithelioid histology and be treated in an urban setting (*p* = 0.0071 and *p* = 0.0174, respectively). In the adjusted Cox proportional hazards model, delayed TTI was statistically significantly associated with improved survival compared to early TTI (HR_adj_: 0.67, 95% CI: 0.54–0.84) among surgical mesothelioma patients.

For patients with epithelioid histology (*n* = 2192), median overall survival in the early TTI group was 14 months, compared to 16 months in the delayed TTI group (*p* = 0.0398; median TTI of 41 days). The two groups varied significantly only in terms of treatment. A greater percentage of the delayed TTI group received combination therapy, whereas a greater percentage of the early TTI group received systemic therapy only (*p* < 0.0001). After adjustment for confounding, delayed TTI remained associated with better patient survival (HR_adj_: 0.87, 95% CI: 0.79–0.95).

For patients with regional or localized SEER stage (*n* = 1488), median overall survival in the early TTI group was 11.5 months, compared to 14 months in the delayed TTI group (*p* = 0.0167; median TTI of 40 days). Notably, the two groups varied significantly in terms of race (*p* = 0.0173). A greater proportion of early TTI patients were White (83% vs. 77%), while a greater proportion of delayed TTI patients were Black (18% vs. 14%). This impact of race on TTI was only observed in this subsample analysis. Among patients with locoregional disease, delayed TTI was statistically significantly associated with improved survival (HR_adj_: 0.83, 95% CI: 0.74–0.93).

## 4. Discussion

In this study of a large sample of patients with MPM, we observed a paradoxical finding that delayed time to treatment initiation is associated with better survival. Other findings of our analysis—that male sex, Black race, fibrous and biphasic histologies, receipt of monotherapy or a non-surgical combination therapy, and greater stage at diagnosis are associated with poorer overall survival—are consistent with existing literature. Furthermore, the data also reveals a decreasing incidence of MPM in the United States.

However, the finding that delayed TTI is associated with better survival reflects a rather unique case compared to most adult malignancies. To account for potential prognostic confounders that differ between early and delayed TTI patients, we conducted a sensitivity analysis on only patients with epithelioid histology—a form of MPM associated with better survival. In this subset of patients, delayed TTI remained significantly associated with better overall survival (Supplemental Table S3). This finding could be seen again in an additional analysis of only patients with locoregional disease (Supplemental Table S4). Thus, even among specific subgroups of patients with favorable disease characteristics, this surprising relationship between TTI and survival remains evident.

Potential explanations for this result may center around some of the key differentiating characteristics of mesothelioma management that distinguish it from other cancers. Given the rarity of mesothelioma compared to other thoracic malignancies, most oncologists have limited experience with mesothelioma treatment, and the complex level of multimodal care necessary is often only available at particularly high-volume tertiary centers [21]. As a result, patients are often required to travel to distant sites for their treatment—a fact that would reasonably drive higher TTI. This notion is supported by data demonstrating that greater travel distance is positively associated with survival for patients with operable MPM [11].

A second potential explanation for why increased TTI is associated with better survival may stem from proper treatment allocation. This analysis demonstrates that treatment with a combination surgical approach yields significantly better overall survival compared to surgery or systemic therapy only. Identification of which patients may be most amenable to combination therapy, however, requires a proper workup with imaging, staging, and laboratory tests, and frequently a discussion with a multidisciplinary team. For patients with potentially resectable disease, mediastinoscopy and/or endobronchial ultrasound (EBUS)-mediated staging are often performed alongside PET-CT prior to therapy selection [7]. Patients who are found to have resectable disease (e.g., lack of nodal spread, lack of pleural effusion, good cardiovascular function, etc.) are eligible for surgery, while those who are not may receive systemic therapy. In a sub-analysis of only patients with epithelioid histology—independently associated with better outcomes—median survival continued to vary across early and delayed TTI groups. Treatment selection was the only factor that differed significantly among the two groups, with a greater percentage of patients in the delayed TTI group receiving combination therapy (*p* < 0.0001). Appropriate treatment selection may, therefore, contribute to delays in treatment initiation while also optimizing patient survival probability.

The argument for proper treatment allocation may also be understood in the context of the results from the recently published MARS 2 study: a randomized trial testing the value of chemotherapy alone compared to chemotherapy plus extended pleurectomy decortication [22]. Contrary to data used to create current guideline recommendations—as well as the results of the present analysis—MARS 2 reported inferior survival for patients who received surgery and chemotherapy compared to chemotherapy alone. However, a primary critique of the MARS 2 study design was a failure to properly identify suitable candidates for surgical resection. Authors determined resectability purely by CT, omitting standard-of-care steps in pre-treatment evaluation—including PET-CT—thus inappropriately including a considerable percentage of patients with staging considered to be inconsistent with resection [23].

The present analysis has methodological limitations. First, the reporting of treatment information in SEER is irregular. For receipt of chemotherapy, SEER indicates either: “Yes” or “No/Unknown”; for radiation, SEER is more detailed, providing several options of radiation therapy, as well as “No/Unknown”; for surgery, SEER contains site-specific codes indicating the type of operation performed, as well as an option for “Unknown whether surgery was performed.” Compared to SEER-Medicare data—which is built from claims information—the overall sensitivity in SEER is 68% and 80% for the chemotherapy and radiation data, respectively [24]. To account for this, we repeated the above analysis on only the subset of patients who received surgery alone—a group whose treatment data is more reliably reported (Supplemental Table S2). Median survival in the early TTI group was 4 months, compared to 11 months in the delayed TTI group (*p* < 0.0001). In multivariate analysis, delayed TTI among these patients remained strongly associated with better overall survival. Therefore, in a subset of SEER patients with reliable treatment data reporting, the primary effect of TTI on overall survival remains significant.

Second, due to the necessary de-identification of patient data in SEER, it is not possible to access specific dates of treatment, and therefore, if a patient was noted to receive multiple treatments, the sequence cannot be predicted (e.g., neoadjuvant chemotherapy, adjuvant radiation). This is further complicated by the fact that the TTI data provided in SEER is defined as the time to initiation of the first treatment, and it is unclear which treatment a patient first received. Additionally, SEER considers active surveillance a treatment option, but whether a patient was initiated on active surveillance is not reported. In selecting an appropriate study sample, we excluded all patients with a TTI of 0 under the assumption that these patients were immediately initiated on active surveillance, and we excluded all patients who were not reported to have received any treatment. While our goal was to include only patients who received chemotherapy, radiation, and/or surgery, the limitations of the data reporting make it impossible to determine if this approach was successful. Additionally, excluding all patients with a TTI of 0 could potentially have introduced a degree of selection bias. While there are no clear areas of difference between these patients and the study sample, it remains possible that these patients may have differed in some capacity not reported in SEER.

Third, a key limitation of the SEER dataset is the lack of detailed comorbidity and patient fitness information, so the impacts of these and other unmeasured confounding variables could not be determined. It is also not possible to assess which patients had access to specialized mesothelioma care nor what workup was completed prior to treatment initiation. A follow-up study with SEER-Medicare data, which contains some of these data, is warranted to explore the confounding factors in greater detail.

While the results of this analysis are limited in their generalizability due to sampling restrictions, they may have important implications for clinical practice. Our findings reinforce the critical need for a comprehensive staging workup for all mesothelioma patients before treatment selection. Furthermore, they support the notion that patients are best served by receiving care at high-volume, specialized mesothelioma centers of excellence. Although these steps might delay the start of treatment, our results suggest that such delays are not necessarily linked to poorer survival outcomes. In fact, ensuring that patients undergo thorough staging and receive care tailored to their specific case could lead to improved outcomes.

However, disparities in socioeconomic status, access to healthcare, health literacy, and the ability to travel present significant barriers to implementing these recommendations. Future research should focus on evaluating the costs associated with a complete staging workup and travel to better understand how these factors might limit patients’ ability to access optimal treatment. Additional studies could also explore how mesothelioma differs from other cancers, which may provide further insight into this paradoxical result and inform strategies for improving care for mesothelioma patients.

## 5. Conclusions

This study underscores a unique point in the management of malignant pleural mesothelioma: although treatment should be initiated in a timely manner, a delay in initiation is not necessarily associated with poorer overall survival. Rather, these results suggest that patient travel to mesothelioma centers of excellence—and proper workup of patients once they arrive—should be encouraged for optimal treatment allocation and outcomes.

## Figures and Tables

**Table 1:** Associations Between Demographic, Disease, and Treatment Characteristics and Time to Treatment Initiation (TTI).

Patient Characteristics	Early TTI *n* = 2428, 50%	Delayed TTI *n* = 2451, 50%	*p*
Overall Survival, median (months)	10 months	13 months	<0.0001
Age, mean (years)	70.2	70.1	0.7463
Sex			0.2895
Female	528 (22%)	565 (23%)	
Male	1900 (78%)	1886 (77%)	
Race			0.2274
White	1945 (80%)	1916 (78%)	
Black	369 (15%)	415 (17%)	
Other/Unknown	114 (5%)	120 (5%)	
Income Level			0.3309
<60,000	323 (13%)	299 (12%)	
60,000–99,999	1656 (68%)	1718 (70%)	
>100,000	449 (19%)	434 (18%)	
Marital Status			0.5020
Married	1683 (69%)	1689 (69%)	
Single	654 (27%)	683 (28%)	
Other/Unknown	91 (4%)	79 (3%)	
Stage			0.0351
Localized	205 (8%)	223 (9%)	
Regional	504 (21%)	556 (23%)	
Distant	1652 (68%)	1629 (66%)	
Unknown/Unstaged	67 (3%)	43 (2%)	
Histology			<0.0001
Mesothelioma, NOS	898 (23%)	813 (15%)	
Fibrous (Sarcomatoid)	284 (7%)	221 (6%)	
Epithelioid	1016 (26%)	1176 (38%)	
Biphasic (Mixed)	230 (13%)	241 (10%)	
Disease Laterality			0.0876
Left	930 (38%)	969 (40%)	
Right	1389 (57%)	1401 (57%)	
Bilateral/Unknown	109 (4%)	81 (3%)	
Type of Treatment Received			<0.0001
Surgery Only	220 (9%)	172 (7%)	
Systemic only	1672 (69%)	1701 (69%)	
Combination Therapy	536 (22%)	578 (24%)	
Practice Setting			0.0657
Urban	2001 (82%)	2069 (84%)	
Suburban/Rural	427 (18%)	382 (16%)	
Year of Diagnosis			0.0001
2004–2008	595 (25%)	527 (22%)	
2009–2013	762 (31%)	698 (28%)	
2014–2018	755 (31%)	818 (33%)	
2019–2022	316 (13%)	408 (17%)	

**Table 2:** Association Between TTI and Overall Survival.

Patient Characteristics	HR_adj_ (95% CI) ^1^
TTI Class	
Early TTI	1.0 (Ref)
Delayed TTI	0.79 (0.74–0.84)
Age	1.02 (1.01–1.02)
Sex	
Male vs. Female	1.19 (1.10–1.28)
Race	
White	1.0 (Ref)
Black	1.12 (1.03–1.22)
Other	1.06 (0.92–1.22)
Income	
<$60,000	1.0 (Ref)
$60,000–$99,999	1.01 (0.90–1.12)
>$100,000	0.97 (0.85–1.10)
SEER Stage	
Localized	1.0 (Ref)
Regional	1.16 (1.03–1.32)
Distant	1.39 (1.24–1.56)
Unknown/Unstaged	1.12 (0.90–1.40)
Histology	
Mesothelioma, NOS	1.0 (Ref)
Epithelioid	0.78 (0.73–0.84)
Fibrous	1.83 (1.65– 2.04)
Biphasic	1.26 (1.13–1.40)
Treatment	
Surgery Only	1.0 (Ref)
Systemic Treatment Only	0.97 (0.86–1.08)
Combination Treatment	0.62 (0.54–0.70)
Practice Setting	
Suburban/Rural vs. Urban	1.01 (0.91–1.11)
Year of Diagnosis	0.98 (0.97–0.99)

^1^ Variables adjusted for all others reported in the table.

## Data Availability

Data was harvested from the National Cancer Institute’s Surveillance, Epidemiology, and End Results (SEER) database, accessible at https://seer.cancer.gov/.

## Supplementary Materials

The following supporting information can be downloaded at https://www.mdpi.com/article/10.3390/cancers16223755/s1, Table S1: Overview of Sample Demographic, Disease, and Treatment Characteristics; Table S2: Sensitivity Analysis on Patients Receiving Only Surgery; Table S3: Sensitivity Analysis on Only Patients with Epithelioid Histology; Table S4: Sensitivity Analysis on Only Patients with Locoregional Disease.
