# Supplementary material for: Paradoxical Improvement in Malignant Pleural Mesothelioma Outcomes Following Delayed Treatment Initiation
Source: Cancers (Basel). 2024 Nov 7;16(22):3755. doi: 10.3390/cancers16223755 (PMC11592216; doi:10.3390/cancers16223755)
Supplement: Supplementary file 1 [file cancers-16-03755-s001.zip › cancers-3275523-supplementary.pdf]

Supplemental Data

Supplemental Table S1: Overview of Sample Demographic, Disease, and Treatment Characteristics (n = 4,879)

Patient Characteristics	n (%)	Median TTI (days)	Median OS (months)
**All Patients**	4879	39.0	11
**Age**, mean (years)	70.11		
**Sex**			
Female	1093 (22%)	40.0	13
Male	3786 (78%)	38.0	11
**Race**			
White	3861 (79%)	38.0	11
Black	784 (16%)	41.0	11
Other/Unknown	234 (5%)	39.5	11
**Income Level**			
<60,000	622 (13%)	36.0	11
60,000–99,999	3374 (69%)	39.0	11
>100,000	883 (18%)	38.0	12
**Marital Status**			
Married	3372 (69%)	39.0	12
Single	1337 (27%)	40.0	10
Other/Unknown	170 (3%)	37.0	12.5
**SEER Stage**			
Localized	428 (9%)	39.0	14
Regional	1060 (22%)	40.5	12
Distant	3281 (67%)	38.0	11
Unknown/Unstaged	110 (2%)	34.0	11
**Histology**			
Mesothelioma, NOS	1711 (35%)	36.0	10
Fibrous (Sarcomatoid)	505 (10%)	34.0	6
Epithelioid	2192 (45%)	41.0	15
Biphasic (Mixed)	471 (10%)	40.0	11
**Disease Laterality**			
Left	1899 (39%)	39.0	12

Right	2790 (57%)	39.0	11
Bilateral/Unknown	190 (4%)	31.0	9
**Type of Treatment Received**			
Surgery Only	392 (8%)	34.5	7
Systemic Only	3373 (69%)	39.0	10
Combination Therapy	1114 (23%)	40.0	18.5
**Practice Setting**			
Urban	4070 (83%)	39.0	11
Suburban/Rural	809 (17%)	36.0	11
**Year of Diagnosis**			
2004-2008	1122 (23%)	36.0	11
2009-2013	1460 (30%)	36.0	11
2014-2018	1573 (32%)	40.0	12
2019-2022	724 (15%)	42.0	10

Supplemental Table S2a: Sensitivity Analysis on Patients Receiving Only Surgery (n = 392)

Patient Characteristics	Median OS (months)	Early TTI n = 196, 50%	Delayed TTI n = 196, 50%	p
**All Patients**		196 (50%)	196 (50%)	
Overall Survival, median (months)	7 months	4 months	11 months	**0.0003**
**Age**, mean (years)		73.5 years	69.1 years	**<0.0001**
**Sex**				**1.0000**
Female	6	41 (21%)	42 (21%)	
Male	7	155 (79%)	154 (79%)	
**Race**				**0.4788**
White	8	158 (81%)	167 (85%)	
Black	5	27 (14%)	21 (11%)	
Other	6	11 (6%)	8 (4%)	
**Income Level**				**0.0734**
<60,000	6	27 (14%)	22 (11%)	
60,000 - 99,999	8	143 (73%)	131 (67%)	
>100,000	11	26 (13%)	43 (22%)	
**Marital Status**				**0.3782**
Married	7	116 (59%)	129 (66%)	
Single	7	69 (35%)	59 (30%)	
Other/Unknown	7	11 (6%)	8 (4%)	
**SEER Stage**				**0.1656**
Localized	17	27 (14%)	20 (10%)	
Regional	7	49 (25%)	51 (26%)	
Distant	6	120 (61%)	121 (62%)	
Unknown/Unstaged	42	0 (0%)	4 (2%)	
**Histology**				**0.0071**
Mesothelioma, NOS	8	65 (33%)	44 (22%)	
Fibrous (Sarcomatoid)	4.5	21 (11%)	16 (8%)	
Epithelioid	15	74 (38%)	108 (55%)	
Biphasic (Mixed)	8	36 (18%)	28 (14%)	
**Disease Laterality**				**0.8152**
Left	5	68 (35%)	74 (38%)	
Right	5	119 (61%)	113 (58%)	

Bilateral/Unknown	10	9 (5%)	9 (5%)	
**Practice Setting**				**0.0174**
Urban	7	161 (82%)	167 (85%)	
Suburban/Rural	5.5	35 (18%)	29 (15%)	
**Year of Diagnosis**				**0.7936**
2004-2008	11.5	55 (28%)	53 (27%)	
2009-2013	8	65 (33%)	59 (30%)	
2014-2018	6	52 (27%)	54 (28%)	
2019-2022	5	24 (12%)	30 (15%)	

Supplemental Table S2b: Association Between TTI and Overall Survival - Patients Receiving Only Surgery (n = 392)

Patient Characteristics	HR_{adj} (95% CI)[1]
**TTI Class**	
Early TTI	1.0 (Ref)
Delayed TTI	0.67 (0.54 – 0.84)
**Age**	1.01 (1.00 – 1.02)
**Sex**	
Male vs. Female	1.13 (0.86 – 1.47)
**Race**	
White	1.0 (Ref)
Black	1.07 (0.76 – 1.52)
Other	1.02 (0.61 – 1.68)
**Income**	
<$60,000	1.0 (Ref)
$60,000 - $99,999	1.07 (0.71 – 1.62)
>$100,000	1.05 (0.64 – 1.73)
**SEER Stage**	
Localized	1.0 (Ref)
Regional	1.51 (1.01 – 2.24)
Distant	2.24 (1.56 – 3.24)
Unknown/Unstaged	0.87 (0.30 – 2.56)
**Histology**	
Mesothelioma, NOS	1.0 (Ref)
Epithelioid	0.82 (0.64 – 1.07)
Fibrous	1.91 (1.26 – 2.89)
Biphasic	1.23 (0.88 – 1.73)

**Practice Setting**	
Suburban/Rural vs. Urban	1.15 (0.80 – 1.66)
**Year of Diagnosis**	1.01 (0.99 – 1.04)

[1] Variables adjusted for all others reported in the table

Supplemental Table S3a: Supplemental Analysis on Only Patients with Epithelioid Histology (n = 2192)

Patient Characteristics	Median OS (months)	Early TTI n = 1080, 49%	Delayed TTI n = 1112, 51%	p
**All Patients**		1080 (49%)	1112 (51%)	
Overall Survival, median (months)	15	14 months	16 months	**0.0398**
**Age**, mean (years)		68.9 years	69.5 years	**0.1818**
**Sex**				**0.2895**
Female	18	280 (26%)	271 (24%)	
Male	14	800 (74%)	841 (76%)	
**Race**				**0.9288**
White	15	866 (80%)	887 (80%)	
Black	14	161 (15%)	172 (15%)	
Other/Unknown	17	53 (5%)	53 (5%)	
**Income Level**				**0.0805**
<60,000	14	143 (13%)	117 (11%)	
60,000 - 99,999	15	725 (67%)	790 (71%)	
>100,000	16	212 (20%)	205 (18%)	
**Marital Status**				**0.0828**
Married	15	772 (71%)	746 (67%)	
Single	15	276 (26%)	327 (29%)	
Other/Unknown	15	32 (3%)	39 (4%)	
**SEER Stage**				**0.4338**
Localized	14	99 (9%)	95 (9%)	
Regional	12	246 (23%)	243 (22%)	
Distant	11	711 (66%)	758 (68%)	
Unknown/Unstaged	11	24 (2%)	16 (1%)	
**Disease Laterality**				**0.2003**
Left	15	410 (38%)	638 (57%)	
Right	15	630 (58%)	446 (40%)	
Bilateral/Unknown	12	40 (4%)	28 (3%)	
**Type of Treatment Received**				**<0.0001**
Surgery Only	10	90 (8%)	92 (8%)	
Systemic Only	12	668 (62%)	666 (60%)	

Combination Therapy	22	322 (30%)	354 (32%)	
**Practice Setting**				**0.0554**
Urban	15	885 (82%)	946 (85%)	
Suburban/Rural	14	195 (18%)	166 (15%)	
**Year of Diagnosis**				**0.5647**
2004-2008	15	204 (19%)	210 (19%)	
2009-2013	16	324 (30%)	308 (28%)	
2014-2018	16	384 (36%)	402 (36%)	
2019-2022	12	168 (16%)	192 (17%)	

Supplemental Table S3b: Association Between TTI and Overall Survival - Only Patients with Epithelioid Histology (n = 2192)

Patient Characteristics	HR_{adj} (95% CI)[1]
**TTI Class**	
Early TTI	1.0 (Ref)
Delayed TTI	0.87 (0.79 – 0.95)
**Age**	1.02 (1.01 – 1.02)
**Sex**	
Male vs. Female	1.25 (1.12 – 1.40)
**Race**	
White	1.0 (Ref)
Black	1.17 (1.02 – 1.33)
Other	1.08 (0.88 – 1.34)
**Income**	
<$60,000	1.0 (Ref)
$60,000 - $99,999	0.99 (0.84 – 1.17)
>$100,000	1.01 (0.83 – 1.23)
**SEER Stage**	
Localized	1.0 (Ref)
Regional	1.15 (0.95 – 1.40)
Distant	1.39 (1.17 – 1.66)
Unknown/Unstaged	1.41 (0.98 – 2.04)
**Treatment**	
Surgery Only	1.0 (Ref)
Systemic Treatment Only	1.01 (0.85 – 1.20)
Combination Treatment	0.61 (0.51 – 0.73)
**Practice Setting**	
Suburban/Rural vs. Urban	1.04 (0.90 – 1.20)
**Year of Diagnosis**	0.98 (0.97 – 0.99)

[1] Variables adjusted for all others reported in the table

Supplemental Table S4a: Supplemental Analysis on Only Patients with Locoregional Disease (n = 1488)

Patient Characteristics	Median OS (months)	Early TTI n = 736, 49%	Delayed TTI n = 752, 51%	p
**All Patients**		736 (49%)	752 (51%)	
Overall Survival, median (months)	13	11.5	14	**0.0167**
**Age**, mean (years)		70.9	70.3	**0.2299**
**Sex**				**0.4734**
Female	16.5	158 (21%)	172 (23%)	
Male	12	578 (79%)	580 (77%)	
**Race**				**0.0173**
White	13	608 (83%)	580 (77%)	
Black	12	103 (14%)	133 (18%)	
Other/Unknown	18	25 (3%)	39 (5%)	
**Income Level**				**0.5120**
<60,000	12	89 (12%)	76 (10%)	
60,000 - 99,999	13	511 (69%)	531 (71%)	
>100,000	14	136 (18%)	145 (19%)	
**Marital Status**				**0.6788**
Married	14	504 (68%)	526 (70%)	
Single	11	203 (28%)	199 (26%)	
Other/Unknown	13	29 (4%)	27 (4%)	
**SEER Stage**				**0.9799**
Localized	11	217 (29%)	228 (30%)	
Regional	7	98 (13%)	90 (12%)	
Distant	17	339 (46%)	344 (46%)	
Unknown/Unstaged	12	82 (11%)	90 (12%)	
**Disease Laterality**				**0.6724**
Left	14	296 (40%)	305 (41%)	
Right	12	427 (58%)	439 (58%)	
Bilateral/Unknown	13	13 (2%)	8 (1%)	
**Type of Treatment Received**				**0.0728**
Surgery Only	9	84 (11%)	63 (8%)	
Systemic Only	11	464 (63%)	480 (64%)	

Combination Therapy	20	188 (26%)	209 (28%)	
**Practice Setting**				**0.0638**
Urban	13	609 (83%)	647 (86%)	
Suburban/Rural	11.5	127 (17%)	105 (14%)	
**Year of Diagnosis**				**0.0007**
2004-2008	11	137 (19%)	114 (15%)	
2009-2013	12	195 (26%)	151 (20%)	
2014-2018	16	250 (34%)	311 (41%)	
2019-2022	11	154 (21%)	176 (23%)	

Supplemental Table S4b: Association Between TTI and Overall Survival - Only Patients with Locoregional Disease (n = 1488)

Patient Characteristics	HR_{adj} (95% CI)[1]
**TTI Class**	
Early TTI	1.0 (Ref)
Delayed TTI	0.83 (0.74 – 0.93)
**Age**	1.02 (1.01 – 1.03)
**Sex**	
Male vs. Female	1.30 (1.13 – 1.50)
**Race**	
White	1.0 (Ref)
Black	1.13 (0.96 – 1.33)
Other	0.81 (0.61 – 1.08)
**Income**	
<$60,000	1.0 (Ref)
$60,000 - $99,999	0.87 (0.71 – 1.06)
>$100,000	0.84 (0.66 – 1.07)
**Histology**	
Mesothelioma, NOS	1.0 (Ref)
Epithelioid	0.80 (0.69 – 0.91)
Fibrous	1.91 (1.58 – 2.30)
Biphasic	1.37 (1.13 – 1.66)
**Treatment**	
Surgery Only	1.0 (Ref)
Systemic Treatment Only	1.33 (1.09 – 1.62)
Combination Treatment	0.80 (0.65 – 1.00)
**Practice Setting**	
Suburban/Rural vs. Urban	1.10 (0.91 – 1.32)

**Year of Diagnosis**	0.97 (0.95 – 0.98)

[1] Variables adjusted for all others reported in the table
